# Opsonizing antibodies mediated SARS‐CoV‐2 entry into monocytes leads to inflammation

**DOI:** 10.1002/mef2.11

**Published:** 2022-07-25

**Authors:** Tong Yao, Shuai Wang, Long Zhang, Johnson Yiu‐Nam Lau, Fangfang Zhou

**Affiliations:** ^1^ Institutes of Biology and Medical Science Soochow University Suzhou Jiangsu China; ^2^ MOE Laboratory of Biosystems Homeostasis and Protection, Innovation Center for Cell Signaling Network Zhejiang University Hangzhou Zhejiang China; ^3^ Department of Biology Hong Kong Baptist University Kowloon Tong Hong Kong China

A recent paper published in *Nature* by Caroline Junqueira et al. (2022)^1^ reveals that FcγR and opsonizing antibodies can mediate severe acute respiratory syndrome coronavirus 2 (SARS‐CoV‐2) infection of monocytes/macrophages, which can then activate NLRP3 inflammasomes, caspase‐1, and Gasdermin D (GSDMD), and thereby trigger pyroptosis and severe inflammatory reaction.

Since the first description in late 2019, coronavirus disease 2019 (COVID‐19), caused by RS‐CoV‐2, has emerged as one of the most significant global public health crises. In some patients, SARS‐CoV‐2 infection can induce a severe inflammatory cytokine storm that can lead to respiratory syndrome and multiorgan failure.^1^, ^2^ From a biological perspective, when myeloid cells sense invasive infection, they activate inflammasomes, which recruit apoptotic speck protein containing a caspase recruitment domain (ASC) adaptors and further activate downstream caspase‐1, which cleaves the suppressant C‐domain and releases the pore‐forming N‐domain of GSDMD (a pyroptosis execution protein that can be activated by caspase‐1), leading to cell membrane breaks, pyroptosis, and the release of inflammatory cytokines^3^ (Figure 1).

**Figure 1:**
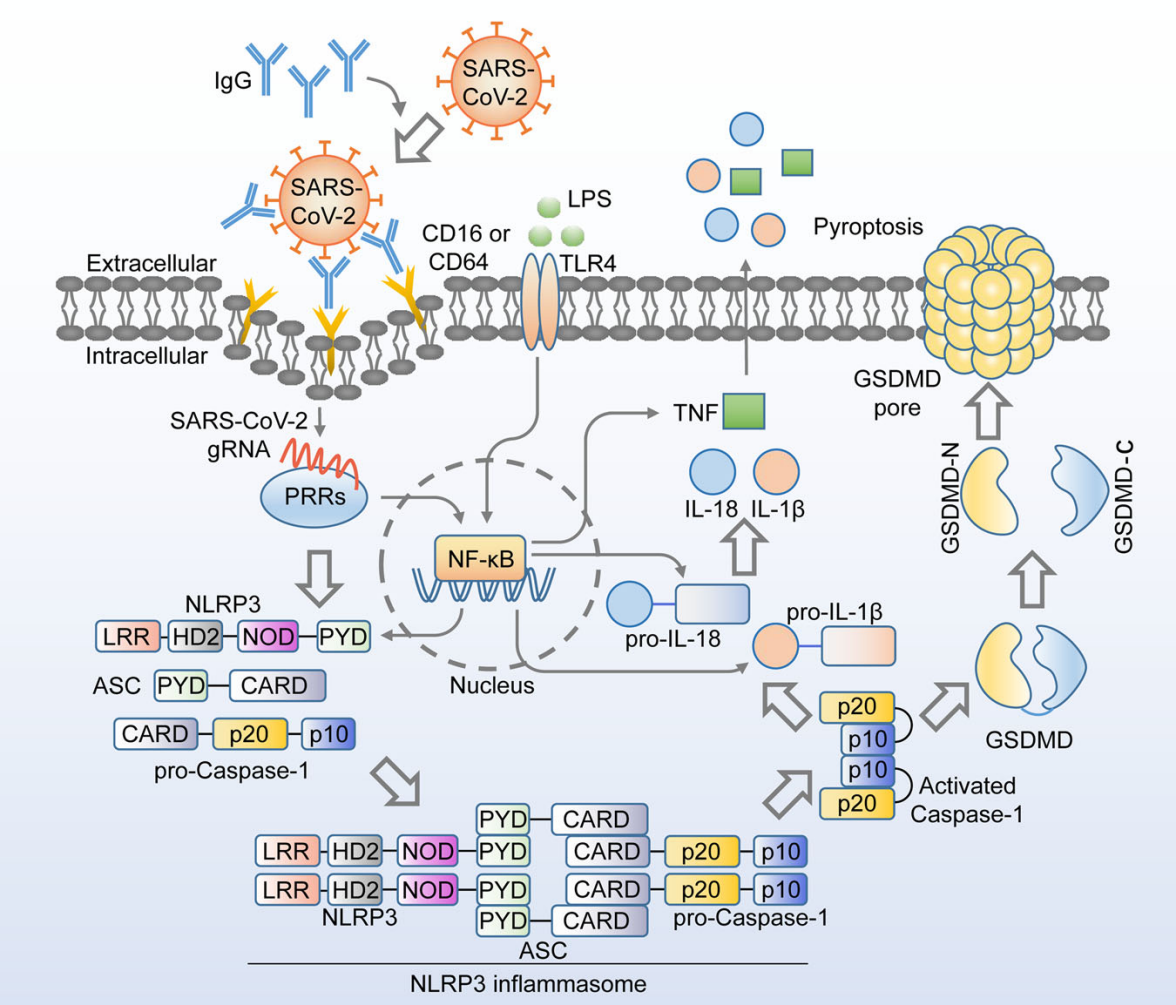
When SARS‐CoV‐2 infects monocytes, NLRP3, ASC, and pro‐caspase‐1 form inflammasome complexes, which subsequently activates caspase‐1. Caspase‐1 can then activate IL‐1β and IL‐18, and cleave the GSDMD C‐domain to release the activated GSDMD‐N domain, which forms a GSDMD pore to destroy the cell membrane, leading to pyroptosis and cytokine release. GSDMD, Gasdermin D; IL, interleukin; SARS‐CoV‐2, severe acute respiratory syndrome coronavirus 2.

In this paper, Junqueira et al.^1^ reported that SARS‐CoV‐2 can also infect monocytes/macrophages, and this is through the Fcγ receptors‐mediated uptake of antibody‐opsonized virus. They then showed that SARS‐CoV‐2 triggers NLRP3 inflammasomes, caspase‐1, and GSDMD‐dependent pyroptosis, an inflammation‐induced programmed cell death,^4^ exacerbating systemic inflammation and the symptoms of COVID‐19. The pyroptosis blocks the infectious virus production despite the replication of the viral genome in monocytes.

The study began with the detection of inflammasome activation and pyroptosis in blood samples obtained from SARS‐CoV‐2‐infected patients. They found that approximately 6% of circulating monocytes from SARS‐CoV‐2 infected patients were stained by Zombie dye, a sign of membrane damage consistent with pyroptosis. A series of pyroptosis biomarkers, including GSDMD, interleukin 1β (IL‐1β), IL‐1RA, IL‐18, and lactate dehydrogenase activity, were studied and found to be elevated. Through imaging flow cytometry, they found that about 4% of monocytes from COVID‐19 patients exhibit activation of canonical inflammasomes, which form large micron‐sized inflammasome‐ASC‐caspase‐1 specks. ASC specks were colocalized with caspase‐1, NLRP3 (a canonical inflammasome), and AIM2. Moreover, the lung‐resident macrophages were also found to contain activated inflammasomes.^1^ Previous studies have proven that several SARS‐CoV‐2 proteins are involved in NLRP3 inflammasome activation. Nucleocapsid (N) protein directly interacts with NLRP3 and contributes to severe inflammatory responses in patients.^5^, ^6^ Spike (S) protein was also proven to activate NLRP3 inflammasome‐induced pyroptosis in hematopoietic stem/progenitor cells.^7^

Given that ACE2, the viral entry receptor, is undetected in monocytes, they examine how SARS‐CoV‐2 activates inflammasome COVID‐19 monocytes. They found that 10% of the monocytes could be stained with SARS‐CoV‐2 N protein and double‐stranded RNA antibody, and the proportion was consistent with the proportion of monocytes expressing CD16 (FcγRIIIa) in blood monocytes (∼10%) (more details on this later).^8^ Moreover, all monocytes with ASC specks were infected; and all infected monocytes had ASC specks, indicating that SARS‐CoV‐2 directly infects monocytes and activates inflammasomes and pyroptosis.

Using an engineered infectious clone (icSARS‐CoV‐2‐mNG, which encodes a neon green (NG) fluorescent reporter of viral replication), the author found that preincubation with antispike monoclonal antibodies or COVID‐19 patient plasma permits the viral entry into monocytes. The highest infection rate requires preincubation of the virus with patient plasma and pretreating of the healthy donor monocytes with lipopolysaccharides. Meanwhile, immunoglobulin G (IgG)‐depletion of COVID‐19 plasma, but not IgA depletion, abolished the viral infection. Purified IgG from COVID‐19 plasma also facilitated viral infection, while IgG from healthy donor plasma barely affect viral entry, suggesting that the infection is mediated by antispike antibody‐opsonized virus. Interestingly, patient plasma potentiated the healthy donor monocyte infection more than purified IgG from COVID‐19 plasma, suggesting that other components might also be involved. Importantly, plasma from vaccinated individuals did not facilitate the viral infection.

To identify the viral receptor, blocking antibodies to ACE2, CD147, CD16, CD32, and CD64, were utilized to block potential monocyte receptors. Blocking of monocyte FcγRs, CD16 or CD64, drastically inhibited infection, suggesting that CD16 or CD64 mediate the entry of opsonized SARS‐CoV‐2 into monocytes. Although blocking antibodies to CD64 could also block the infection, CD64 is more widely expressed than CD16, that is, more restricted and as all infected monocytes expressed CD16; the authors concluded that CD16 is probably the major Fc receptor. Despite neutrophils, cytotoxic T and NK cells also express CD16, SARS‐CoV‐2 infection was undetected in these cells of COVID‐19 patients, implying that the antibody‐dependent entry is confined to monocytes. Furthermore, despite quantitative polymerase chain reaction assays confirming the replication of the viral genome, no infectious virus was detected in SARS‐CoV‐2‐infected healthy donor monocyte culture supernatants, indicating that pyroptosis and inflammatory responses terminate the productive viral cycle.

This paper showed the following important points. First, they confirmed the antibody‐mediated SARS‐CoV‐2 infection of monocytes. The antibody‐mediated enhancement of infection has been reported in serval viruses, including influenza virus, respiratory syncytial virus, Middle East respiratory syndrome coronavirus, SARS‐CoV, as well as dengue virus. Similarly, a recent study reported that the monoclonal antibodies of SARS‐CoV‐2 S protein facilitate the SARS‐CoV‐2 entry into lung‐resident macrophages, driving inflammasome activation and pyroptosis. Blocking CD16 significantly restrained the SARS‐CoV‐2 entry despite macrophages also expressing ACE2. Caspase‐1 and NLRP3 inhibitors ameliorate inflammation but lead to the release of infectious viruses from the infected macrophages.^9^ Another study showed that IgG receptors, FcγRIIA and FcγRIIIA, can contribute to antibody‐dependent enhancement of SARS‐CoV‐2.^10^

Second, this study reveals that the infection of monocytes can induce pyroptosis of monocytes, releasing the inflammatory cytokines and contributing to the pathogenesis of COVID‐19. Pyroptosis might act as a double‐edged sword during SARS‐CoV‐2 infection, which on the one hand eliminates viral infection before producing infectious virions, and on the other, facilitates the release of inflammatory factors contributing to the cytokine storm. These findings may provide some insights into the pathogenetic mechanism involved that some patients had clinical deterioration, which coincides temporally with the detection of the SARS‐CoV‐2 antibody.

Finally, and fortunately, the antibodies induced by the COVID‐19 vaccines do not mediate the infection, probably related to the binding to different epitopes, or related to the change in a typology that did not facilitate the viral entry.

## AUTHOR CONTRIBUTIONS

Tong Yao and Shuai Wang wrote the manuscript; Long Zhang, Johnson Yiu‐Nam Lau, and Fangfang Zhou provided valuable discussion.

## CONFLICT OF INTEREST

The authors declare no conflict of interest.

### ETHICS STATEMENT

Not applicable.

## Data Availability

Not applicable.
